# Effects of dietary aflatoxin B_1_ on accumulation and performance in matrinxã fish (*Brycon cephalus*)

**DOI:** 10.1371/journal.pone.0201812

**Published:** 2018-08-08

**Authors:** Carolina M. Bedoya-Serna, Euder C. Michelin, Marina M. Massocco, Lucas C. S. Carrion, Silvia H. S. Godoy, Cesar G. Lima, Paulo S. Ceccarelli, George S. Yasui, George E. Rottinghaus, Ricardo L. M. Sousa, Andrezza M. Fernandes

**Affiliations:** 1 Department of Veterinary Medicine, Faculty of Animal Science and Food Engineering, University of São Paulo - USP, Pirassununga, São Paulo, Brazil; 2 Department of Basic Sciences, Faculty of Animal Science and Food Engineering, University of São Paulo - USP, Pirassununga, São Paulo, Brazil; 3 Chico Mendes Institute for Biodiversity Conservation - ICMBio, Pirassununga, São Paulo, Brazil; 4 Veterinary Medical Diagnostic Laboratory, College of Veterinary Medicine, University of Missouri, Columbia, Missouri, United States of America; University of Illinois, UNITED STATES

## Abstract

Aflatoxins (AF) can be cumulative in fish tissues and can influence weight, length, feed intake and survival depending on the species. The aim of this work is to measure performance and aflatoxin levels in tissues of matrinxã (*Brycon cephalus*) fish chronically exposed to aflatoxin. Aflatoxin was incorporated into fish diets at the following levels: Control Feed + 0 μg AFB_1_ kg^-1^; A. Feed + 10 μg AFB_1_ kg^-1^; B. Feed + 20 μg AFB_1_ kg^-1^; C. Feed + 50 μg AFB_1_ kg^-1^. It was used one tank per treatment, each one with 150 juvenile fish, and three replicates within each tank were used for sampling, that was carried out monthly over a period of six months. Aflatoxin was quantified by HPLC in fish liver and muscle after clean up using immunoaffinity columns. Performance was evaluated by using weight, length, consumption and survival rate. Muscle and liver aflatoxin levels were below the limit of detection in all control samples. Aflatoxins B_2_, G_1_ and G_2_ were not detected in any tissues. Traces (values between limits of detection and quantification) of AFB_1_ were observed in liver tissue in treatment A from day 30 through 90, reaching 0.32 μg AFB_1_ kg^-1^ at 150 days of exposure. Treatment B presented traces up to day 60 and had, with a maximum level of 0.39 μg AFB_1_ kg^-1^ at 150 days of exposure. Treatment C had aflatoxin residues after day 30, with values ranging from 0.17 to 0.61 μg AFB_1_ kg^-1^ during exposure. Muscle samples only had traces of AFB_1_ in all treatments. Fish was affected by exposure to AFB_1_ with higher values (P<0.05) for weight and length in treatments A, B and C relative to controls. Therefore, results indicate that matrinxã do not accumulate AFB_1_ residues in edible tissues, but chronic exposure affects the species.

## Introduction

In the last 20 years there has been a dramatic increase in commercial fish production, leading to an increase in the average consumption of fish worldwide. Brazil is currently among the ten largest aquaculture producers in the world. Growth in world aquaculture is expected to increase in the coming years, with Brazil leading the way in Latin America with projections of a 104% higher production in 2025. Moreover, an increase in fish consumption is expected with significant growth in countries such as Brazil, Peru, Chile, China and Mexico [1].

The matrinxã (*Brycon cephalus*) is a teleost fish found in the Brazilian Amazon and it is widely cultivated in Brazil due to its taste and high quality protein [2, 3]. The matrinxã has a silver-colored elongated body with orange fins, dark caudal fin, scales and strong multi-rostral teeth arranged in several rows in the upper jaw. The species can reach 80 cm in length and weigh up to 5 kg [4]. The matrinxã is omnivorous, feeding on seeds, fruits, flowers, plant remains, herbaceous plants, fish remains, arachnids, annelids and insects [5]. According to Gadelha and Araújo [3], the species is very promising for fish farming because it has good feed conversion, easy adaptation to feed, high fecundity and rapid growth. The spawning period is from December to January and the species has excellent performance in tanks, regardless of density [6]. Production of matrinxã in Brazil was 9,366,203 kg in 2015 [7]. It has a fillet carcass yield of approximately 40%, moisture content of 60 to 62%, 2 to 3% minerals, 18% fat and 18 to 19% protein [8].

Aflatoxins (AF) are a major group of mycotoxins with aflatoxin B_1_ (AFB_1_) being the most toxic and considered the highest risk for health [9]. Aflatoxins are carcinogenic, mutagenic, teratogenic and immunosuppressive and are commonly found in foodstuff worldwide [10]. The International Agency for Research on Cancer (IARC) classifies aflatoxins as Group 1 –carcinogenic to humans [11].

The presence of aflatoxin in the feedstuff is harmful to animals and to humans who consume animal derived products contaminated with aflatoxin residues [12]. Cereals are the main feed ingredients that are sources of fungi and mycotoxins for fish and consequently for humans [13].

The residue levels of aflatoxin in fish occur in muscle and liver [14]. Differences in aflatoxin sensitivity in fish species, is due to differences in metabolism among species [15, 16, 17]. In Brazil, Lopes et al. [18] reported residual levels of aflatoxin in the liver and carcass of jundiá (*Rhamdia quelen*) exposed to concentrations of aflatoxin higher than 350 μg kg^-1^ in the feed. Michelin *et al*. [19] studied lambari (*Astyanax altiparanae*) fish and found residues of aflatoxin in liver and muscle after 90 days of exposure to 50 μg AFB_1_ kg^-1^ and after 120 days fish had levels of AFB_1_ in the muscle similar to the levels found in the diet.

The aflatoxins in contaminated diets are responsible for causing damage to the species in fish farming, decreasing growth. Thus, they cause economic losses, reducing productive performance and causing fish mortalities [18]. Abdelhamid [17] stated that the metabolism of aflatoxin is different for each species of fish. The physiology and performance of some species of fish are more sensitive to the effects of aflatoxins. Similar to what happens with the carryover of aflatoxin levels in fish tissues, performance varies depending on the species [17].

The limit for aflatoxin in animal feed recommend by Brazilian regulations is 50 μg kg^-1^ [20] and there is no differentiation among species. In the United States, the limit for aflatoxin is 20 μg kg^-1^ in corn, cereals and cereal-based products; and in Europe the limit for aflatoxin in feed for livestock production is 10 μg kg^-1^ [21]. Most studies available in the literature have evaluated the effects of aflatoxins at high levels in the diet; however, the effects of diets contaminated with aflatoxins at allowed levels are unclear, especially in fish. Besides, the aflatoxins limits are established for all animals, even the species being completely different. Therefore, the aim of this study was to verify the carryover of aflatoxin B_1_ from feed to liver and muscle and evaluate the effects on weight, length, feed intake and survival of matrinxã fish.

## Materials and methods

### Aflatoxins production

Aflatoxins were produced (528 μg AFB_1_ g^-1^) in culture according to the methodology described by Shotwell et al. [22], using *Aspergillus parasiticus* NRRL (Northern Regional Research Laboratory) 2999 from Agriculture Research Service (ARS) culture collection, United States Department of Agriculture.

The commercial feed (Laguna peixes tropicais, Socil, Descalvado, Brasil) components and the levels of guarantee are presented in Table 1. Contaminated diets were prepared by extracting the aflatoxins from the culture material with methanol:water (80:20), stirred, filtered and diluted. The diets were immersed in methanol:water (70:30), left overnight and dried at 60°C for 10 h in a forced air oven as described by Michelin et al. [19], to reach the initial moisture content. The levels of aflatoxins were checked by high performance liquid chromatography (HPLC), as described in the next section for feed, and diets were stored at -80°C until using.

**Table 1:** Feed components and levels of guarantee.

Composition	Guarantee levels per kg	Composition	Guarantee levels per kg
Crude protein (min)	320 g	Vitamin A (min)	12000 UI
Ethereal extract (min)	50 g	Vitamin D3 (min)	2400 UI
Crude fiber (max)	50 g	Vitamin E (min)	50 UI
Minerals (max)	140 g	Vitamin K3 (min)	5 mg
Calcium (min)	3 g	Vitamin B1 (min)	10 mg
Calcium (max)	15 g	Vitamin B2 (min)	20 mg
Phosphorus (min)	6 g	Niacin (min)	100 mg
Sodium (min)	3 g	Pantothenic acid (min)	50 mg
Iron (min)	30 mg	Vitamin B6 (min)	10 mg
Cupper (min)	5 mg	Folic acid (min)	4 mg
Zinc (min)	60 mg	Biotin (min)	0,1 mg
Manganese (min)	30 mg	Vitamin B12 (min)	40 mg
Selenium (min)	0,3 mg	Vitamin C (min)	270 mg
Cobalt (min)	0,1 mg	Moisture	120 g
Iodine (min)	1 mg		

Ingredients: corn gluten meal, ground whole corn, wheat bran, meat and bone meal, hydrolyzed feather meal, calciric limestone, sodium chloride, kaolin, iron sulfate, copper sulfate, manganese monoxide, zinc oxide, cobalt sulfate, calcium iodate, sodium selenite, vitamin A, vitamin D_3_, vitamin E, vitamin K_3_, vitamin B_1_, vitamin B_2_, niacin, pantothenic acid, vitamin B_6_, folic acid, biotin, vitamin B_12_, vitamin C, ethoxyquin, butyl hydroxyanisole (BHA), propionic acid and ammonium hydroxide.

### Experimental conditions

Treatments were defined as: Control − Feed + 0 μg AFB_1_ kg^-1^; A. Feed + 10 μg AFB_1_ kg^-1^; B. Feed + 20 μg AFB_1_ kg^-1^ and C. Feed + 50 μg AFB_1_ kg^-1^, according to the regulatory levels allowed in European Union, United States of America and Brazil, respectively.

The experiment was carried out for 180 days. Matrinxã fish (*Brycon cephalus*) were placed in tanks at Chico Mendes Institute for Biodiversity Conservation and all analyses were performed at Faculty of Animal Science and Food Engineering of University of Sao Paulo (FZEA/USP), both located in Pirassununga, Sao Paulo, Brazil.

Juvenile fish approximately 10–20 cm long were placed in four tanks of 1m^3^ capacity (one tank filled with 800 L of water per treatment and 150 fish per tank) for 21 days before beginning the experiment. Fish were fed twice daily with floating extruded feed, at 5% of animal biomass per day.

This experiment was conducted in accordance with ethical principles, and was approved by the Research Ethics Committee of the Faculty of Animal Science and Food Engineering, University of São Paulo (FZEA/USP), protocol no. 15.1.145.74.9. Fish were daily monitored and before sampling at 30, 60, 90, 120, 150 and 180 days of experiment, fish were fasted for 24 hours. Fish were captured using mesh dip nets (1 mm) and immediately euthanized by immersing in glass aquaria (50 L) with water containing benzocaine hydrochloride 250 mg L^-1^. The euthanasia time was characterized by the observation of total loss of balance, lack of movement of the fins and swimming, reduction in opercular movements and responses only to intense tactile stimuli. For biometry, the fish were anesthetized with 100 mg L^-1^ benzocaine hydrochloride, until cessation of opercular ventilation. After measurements, the fish returned to an aquarium filled with regular water and with intense aeration, to return to the state of regular swimming in physiological position and constant opercular ventilation.

The unit sample was constituted by a pool of approximately five to 10 fish, depending on the size. Aflatoxins were quantified by HPLC in fish muscle and liver after 30, 60, 90, 120, 150 and 180 days of experiment. Each sampling time (once a month) was constituted by 12 samples (a pool of five to 10 fish subsampled three times for each one of the four treatments, taken equally from the three replicate), adding up to 72 liver samples and 72 muscle samples at the end of 180 days. Since the water circulated among the tanks and then the conditions were exactly the same for all treatments, there was no effect of the tank and each subsample was considered as a replicate.

The water was provided from a municipal supply, maintained at 27 ± 1°C and daily checked using a digital pHmeter (Gehaka, model PG 1800, São Paulo, Brazil), a thermocouple thermometer (Comark KM28/P7, Norfolk, UK), Spectro kit for ammonia in fresh water (Cienlab, Campinas, Brazil) and a water quality meter (YSI, model 57, Concord, USA). The pH ranged from 5.0 to 7.1 (mean 5.8), dissolved oxygen from 5.2 to 6.8 (mean 6.0 mg L^-1^) and average total ammonia was 0.3 mg L^-1^. Tanks had regular verification of chlorine and were daily cleaned removing excrement and replacing 70% of water.

### Aflatoxin determination in feed

The main aflatoxins (B_1_, B_2_, G_1_ and G_2_) [23] concentrations were determined in feed samples by HPLC using immunoafinity columns Aflatest WB^®^ (Vicam, Watertown, USA) clean up, as described by Michelin et al. [19]. Briefly, the feed sample and NaCl were added to methanol:water (80: 20), homogenised and filtered. A PBS was added and the combined solution was passed through the immunoafinity column. Aflatoxin was eluted with methanol and the methanol evaporated to dryness, derivatization with trifluoroacetic acid and resuspension in acetronitrile:water (2:8). Quantification of aflatoxins was achieved using an HPLC system Shimadzu Prominence LC-20A (Shimadzu Corporation, Kyoto, Japan) with a fluorescence detection Shimadzu SPD-20A (excitation 350 nm, emission 450 nm) and separation on a HyperClone^®^ 4.6 × 100 mm column (Phenomenex, Torrance, CA, USA), with a pre-column (5 μm) 4 × 10 mm. The mobile phase was water:methanol:acetonitrile (540:100:100) pumped at a flow rate of 1.0 mL min^–1^. Aflatoxin standard mix (AFB_1_, AFB_2_, AFG_1_ and AFG_2_) was 20 μg mL^–1^ in acetonitrile (Oekanal^®^, Sigma Aldrich, St. Louis, MO, USA). Recovery studies were performed in samples spiked with 5 and 20 μg AF kg^-1^ in order to validate the method.

### Aflatoxins determination in fish liver and muscle

Aflatoxins were determined in liver samples by HPLC using immunoafinity column clean up, Aflatest WB^®^ (Vicam, Watertown, USA), as described by Michelin et al. [19]. The method is very similar to that used for feed samples, only differing in the quantities used and a step for filtering the supernatant through a PTFE 0.45 μm membrane.

Aflatoxin concentrations in muscle samples were determined with the same methodology [19], HPLC using immunoafinity column clean up Aflatest WB^®^ (Vicam, Watertown, USA). Recovery assays for tissues were performed as described for feed samples.

### Evaluation of weight, length, feed intake and survival of fish

At the end of each 30-day period, fish was evaluated for weight, length, feed intake and survival rate. Ten fish were used from each tank, with a total of 30 fish per treatment.

Fish weight was measured individually on a precision scale (Acculab^®^ V-1200, New York, USA). The length was measured with a digital calliper (Starfer Digital 150 X 0.02, Brazil), from the anterior part of the head to the end of the caudal fin. Feed consumption was calculated from the amount of feed offered daily and the amount of remaining. The leftovers were collected with a net, about 30 minutes after offering, and were taken to drying at an oven at 60°C for 24-48h to reach the initial moisture followed by weighing. The survival rate was obtained by the difference between the final and initial counts in each tank, considering fish removing during the samplings.

### Analysis of results and ethical aspects

Data were submitted to analysis of variance, using a model of repeated measurements with a treatment factor (level of AFB_1_, with 3 levels) and a longitudinal factor (time of exposure, with 6 levels), in a completely randomized design with three replicates. Proc mixed of SAS 9.3 was used. Means were compared using Tukey test at 5% probability.

This experiment was approved by the Ethical Committee from Faculty of Animal Science and Food Engineering of University of Sao Paulo (FZEA/USP).

## Results and discussion

### Experimental diets

Although *A*. *parasiticus* is able to produce aflatoxins B_1_, B_2_, G_1_ and G_2_ [23], AFB_1_ concentrations were used to establish the treatments and reach the levels intended, as it is the most potent aflatoxin. Therefore, diets were prepared at 10 μg AFB_1_ kg^-1^ (treatment A), 20 μg AFB_1_ kg^-1^ (treatment B) and 50 μg AFB_1_ kg^-1^ (treatment C). Table 2 presents the aflatoxin levels measured in final diets.

**Table 2:** Aflatoxins levels in experimental diets.

Treatment	Aflatoxins levels (μg kg^-1^)				
	AFB_1_	AFB_2_	AFG_1_	AFG_2_	Total AF
Control	ND	ND	ND	ND	ND
A	10.42^c^ ± 3.79*	2.07 ± 2.95	0.87 ± 1.32	1.51 ± 2.27	13.15^c^ ± 5.25
B	25.71^b^ ± 5.62	2.75 ± 2.17	0.56 ± 0.28	0.23 ± 0.20	28.11^b^ ± 6.55
C	56.47^a^ ± 14.04	5.95 ± 3.26	1.18 ± 1.20	0.21 ± 0.13	62.36^a^ ± 16.53

A: 10 μg AFB_1_ kg^-1^; B: 20 μg AFB_1_ kg^-1^; C: 50 μg AFB_1_ kg^-1^.

*Results expressed as mean ± standard deviation. Means followed by different lower case letters in the same column differ significantly (P<0.05).

Limit of detection (LOD): 0.03 μg kg^-1^; Limit of quantification (LOQ): 0.09 μg kg^-1^.

ND: not detected.

### Validation of HPLC method

The retention times for AFG_1_, AFB_1_, AFG_2_ and AFB_2_ were 4.61, 6.92, 10.37 and 16.27 minutes, respectively. The coefficient of determination (R^2^) for AFB_1_, AFB_2_, AFG_1_, AFG_2_ curves were 0.997, 0.997, 0.997 and 0.998, respectively. Recovery values in diets were 86.2% for AFB_1_, 92.9% for AFB_2_, 85.8% for AFG_1_ and 88.4% for AFG_2_. The limits of detection and quantification in diets were 0.03 μg kg^-1^ and 0.09 μg kg^-1^, respectively. The mean recovery for AFB_1_ levels in muscle was 83.1%. The limit of detection in muscle was 0.03 μg kg^-1^ and the limit of quantification was 0.09 μg kg^-1^. The mean recovery for AFB_1_ levels in liver was 104.0%, with limits of detection and quantification of 0.05 μg kg^-1^ and 0.15 μg kg^-1^, respectively.

### Aflatoxins in fish tissues

Aflatoxins B_2_, G_1_ and G_2_ were not detected. The results of AFB_1_ in matrinxã liver are presented in Table 3. AFB_1_ was detected in matrinxã liver over the entire period of exposure; however, the levels were low. Numeric higher levels of AFB_1_ were observed for treatment C when compared to the other treatments on days 30, 60 and 180 of exposure. Aflatoxins B_2_, G_1_ and G_2_ were not detected in matrinxã liver.

**Table 3:** Aflatoxin B_1_ residues in liver and muscle of matrinxã (*Brycon cephalus*) fish daily exposed for 180 days.

Treatment	AFB_1_ (μg kg^-1^)					
	Day 30	Day 60	Day 90	Day 120	Day 150	Day 180
*Liver*						
A	traces	traces	traces	0.18±0.03	0.32±0.03	0.15±0.03
B	traces	traces	0.18±0.04	0.27±0.03	0.39±0.03	traces
C	0.41±0.03*	0.17±0.03	0.26±0.03	0.31±0.04	0.40±0.03	0.61±0.04
*Muscle*						
A	traces	ND	ND	ND	traces	ND
B	ND	ND	ND	ND	traces	ND
C	traces	ND	traces	ND	traces	traces

A: 10.42 μg AFB_1_ kg^-1^; B: 25.71 μg AFB_1_ kg^-1^; C: 56.47 μg AFB_1_ kg^-1^.

*Results expressed as mean ± standard error.

Liver: Limit of detection (LOD): 0.05 μg kg^-1^; Limit of quantification (LOQ): 0.15 μg kg^-1^. Muscle: Limit of detection (LOD): 0.03 μg kg^-1^; Limit of quantification (LOQ): 0.09 μg kg^-1^. ND: not detected. Traces = between LOD and LOQ. All control treatments were ND.

There was no accumulation of AFB_1_ in matrinxã muscle at the doses and period studied, since only trace amounts (<0.09 μg kg^-1^) were detected in several samples (Table 3). The other aflatoxins were not detected in matrinxã muscle.

Due to their liposolubility, the aflatoxins are absorbed in the gastrointestinal tract and distributed to muscle, kidneys, adipose tissue and mainly to liver [24]. In the present work there was not a significant accumulation of aflatoxins in matrinxã tissues, contrary to results found in lambari (*Astyanax altiparanae*) tissues [19]. In fact, studies with several species of fish reported different results, due to the difference in sensitivity and metabolism among species [15, 16, 17].

In several studies, aflatoxin levels were reported in the liver [14, 18, 25] and in the muscle [14, 18, 26, 27, 28, 29, 30] of exposed fish, while in other fish species there was no levels reported in muscle [25, 31, 32]. Several studies evaluated the levels of aflatoxin in fish tissues exposed to dietary aflatoxins.

Hussain et al. [26] reported that walleye fish (*Sander vitreus vitreus*) fed 50 μg kg^-1^ and 100 μg kg^-1^ of aflatoxin for 30 days had levels of AFB_1_, AFB_2_, AFG_1_ and AFG_2_ in muscle at concentrations of 5 μg kg^-1^, 10 μg kg^-1^, 15 μg kg^-1^ and 20 μg kg^-1^, respectively. Deng et al. [25] fed tilapia (*Oreochromis niloticus*) AFB_1_ at levels of 19 μg kg^-1^, 85 μg kg^-1^, 245 μg kg^-1^, 638 μg kg^-1^, 793 μg kg^-1^ and 1641 μg kg^-1^ for 20 weeks. After 15 weeks, aflatoxins were found in the liver from the 85 μg kg^-1^ dose, at concentrations ranging from 10.2 μg kg^-1^ to 24.0 μg kg^-1^. After 20 weeks, liver concentrations ranged from 30.4 μg kg^-1^ to 47.4 μg kg^-1^. There was no accumulation of aflatoxins in the muscle of this species. The authors concluded that tilapia have low susceptibility to AFB_1_.

Rainbow trout (*Oncorhynchus mykiss*) exposed for seven days to a concentration of 219 μg AFB_1_ kg^-1^ had levels of 150 μg AFB_1_ kg^-1^ in muscle. In addition, AFB_1_, AFM_1_ and aflatoxicol levels in muscle were observed in an experiment with fish exposed to 6,276 μg kg^-1^ for seven days, with AFB_1_ varying from 4.1 μg kg^-1^ after 3 hours of feeding to 2.2 μg kg^-1^ after 24 h, AFM_1_ ranging from 0.05 μg kg^-1^ after 3 h and 0.03 after 24 h and aflatoxicol with values of 2.1 μg kg^-1^ to 1.6 μg kg^-1^ after 3 h and 24 h, respectively [28]. Since aflatoxicol levels were higher levels of AFM_1_, the authors suggested that aflatoxicol is the main metabolite of AFB_1_. Nomura et al. [28] also stated that AFB_1_ concentrations in the liver were higher than those found in the muscle. The gibel carp (*Carassius auratus gibelio*) fed aflatoxin for 24 weeks at levels of 22.3 μg kg^-1^ and 1646.5 μg AFB_1_ kg^-1^ had AFB_1_ concentrations of 3.11 μg kg^-1^ at 4.00 μg kg^-1^ in the muscle [30].

In a study carried out in Brazil with jundiá fish (*Rhamdia quelen*), Lopes et al. [18] found residues of AFB_1_ in two experiments. In the first one, with 45 days exposure to diets contaminated with 41 μg kg^-1^, 90 μg kg^-1^ and 204 μg kg^-1^, they found 1.0 μg kg^-1^ and 6.1 μg kg^-1^ in muscle for the highest two levels, respectively. In the second experiment, they used aflatoxin at 350 μg kg^-1^, 757 μg kg^-1^ and 1,177 μg kg^-1^, resulting in aflatoxin residues in muscle of 1.8 μg kg^-1^, 3.1 μg kg^-1^ and 6.7 μg kg^-1^, respectively, and in the liver at 1.6 μg kg^-1^, 4.0 μg kg^-1^ and 12.9 μg kg^-1^, respectively.

The tolerable daily intake of AFB_1_ stipulated by the Food and Drug Administration (FDA) is 5 μg kg^-1^ [33]. This level was reached in the lambari (*Astyanax altiparanae*) muscle after 90 days of exposure, even at treatment levels of 10 μg AFB_1_ kg^-1^ [19]. On the other hand, matrinxã exposed to aflatoxins in the diet for six months appeared to not accumulate aflatoxin in their tissues.

In general, variations among fish species, and in some cases among animals, are due to the metabolism of aflatoxins. Factors influencing the metabolism of aflatoxins include species, sex, age, health status and diet. The efficiency of activation or detoxification in an animal species determines the toxicity of aflatoxins [34].

Santacroce et al. [35] reported that the differences in susceptibility to aflatoxins in aquatic organisms appeared to correlate with interspecies variations in the efficiency of AFB_1_ biotransformation. In some fish species, the metabolic pathways of AFB_1_ are mainly characterized by two routes: phase I, or activation phase, mediated by cytochrome P450; and phase II, or detoxification step, involving two enzymes, glucuronyl transferase and, to a lesser extent, glutathione-S-transferase.

In the channel catfish (*Ictalurus punctatus*), a species considered resistant to aflatoxins, AFB_1_ and its metabolites are rapidly distributed but are not fully absorbed, as indicated by the large amount of AFB_1_ excreted in the faeces. AFB_1_ in this species is rapidly converted to aflatoxicol, which enables rapid elimination. Conversely, in rainbow trout, an aflatoxin sensitive species, all the AFB_1_ appears to undergo epoxidation, activating AFB_1_ in the highly carcinogenic AFB_1_-8,9-epoxide (AFBO) metabolite. The resistance of salmon to aflatoxin can be attributed to the lower efficiency of cytochrome P450-mediated AFB_1_ metabolism for AFBO formation when compared to trout [35].

Thus, in general, the selective sensitivity of fish to AFB_1_ seems to be due to differences in the enzymes involved in the metabolism of AFB_1_. Therefore, different gene expression or enzymatic efficiencies may alter the balance between phases I and II of liver activation and detoxification. Each enzyme, from its expression to its kinetic properties, such as efficiency and affinity, seems to determine resistance or sensitivity to AFB_1_ [35].

Another factor to be considered in the metabolism of aflatoxins in aquatic organisms is the fact that fish fed higher doses of AFB_1_ need to excrete more toxin and therefore it should show a greater ability to eliminate AFB_1_ [29]. This explains in some cases where there are lower liver residues in fish fed higher doses of AFB_1_. Some authors [36] also suggest that the high concentration of AFB_1_ accelerates the biotransformation of the toxin and thus the fish fed high levels of AFB_1_ eliminate the toxin faster than the fish fed lower concentrations.

The presence of mycotoxins in fish feed cannot be neglected, mainly due to their toxic effects and levels of safety in different species. The detection of mycotoxins in fish organs and tissues is essential for estimating the risk to public health and to determine the levels of mycotoxins in different tissues of different species of fish, since levels of tolerance for mycotoxin residues in fish do not yet exist [21]. Specific information on the bioaccumulation of aflatoxins and their metabolites in aquatic organisms in the food chain is necessary for the protection of public health. Therefore, there is a need for studies correlating AFB_1_ levels in the diet with the resulting levels in fish tissue destined for human consumption [35].

### Weight, length, feed intake and survival of fish

The results of matrinxã length and weight are shown in Table 4. There was effect of treatment (P<0.0001) and time of exposure (P<0.0001) for fish length but there was no interaction (P = 0.2393) between the variables. It should be noted that the fish started with similar lengths, grew over time, but from day 60, control treatment fish were significantly larger than the other treatment groups. There was an effect of treatment (P<0.0001) and time of exposure (P<0.0001) for fish weight, in addition to interaction between treatment and time (P = 0.0111). Control treatment fish showed significantly higher weights compared to the other treatment groups after 90 days.

**Table 4:** Length and weight of matrinxã (*Brycon cephalus*) fish daily exposed to aflatoxins for 180 days.

Treatment	Day 0	Day 30	Day 60	Day 90	Day 120	Day 150	Day 180
*Length (cm)*							
Control	15.60±0.35^eA^*	16.29±0.29^deA^	17.33±0.32^cdA^	18.80±0.41^cA^	20.85±0.51^bA^	22.25±0.53^bA^	26.15±0.62^aA^
A	14.61±0.37^eA^	15.28±0.31^deAB^	15.89±0.32^deB^	16.69±0.44^cdB^	18.60±0.59^bcB^	20.19±0.53^bB^	23.82±0.59^aB^
B	14.40±0.35^cA^	14.98±0.29^cB^	14.67±0.32^cC^	17.19±0.44^bB^	18.00±0.56^bB^	20.56±0.53^aAB^	22.60±0.62^aB^
C	14.40±0.35^dA^	15.50±0.31^cdAB^	15.72±0.34^cdBC^	17.00±0.44^cB^	19.38±0.51^bAB^	19.81±0.53^bB^	23.70±0.62^aB^
*Weight (g)*							
Control	50.80±3.03^eA^	63.39±3.37^deA^	79.72±4.59^dA^	107.33±6.77^cA^	162.31±11.22^bA^	190.00±11.56^bA^	282.00±19.93^aA^
A	42.57±3.14^fA^	53.50±3.61^efA^	59.44±4.59^deBC^	80.00±7.27^cdB^	109.70±12.79^bcB^	139.38±11.56^bB^	225.91±19.00^aAB^
B	43.07±3.03^dA^	51.58±3.30^dA^	48.89±4.59^dC^	81.92±7.27^cAB^	110.00±12.19^bcB^	143.75±11.56^abB^	197.50±19.93^aB^
C	45.87±3.03^eA^	57.52±3.53^deA^	66.88±4.86^cdAB^	86.92±7.27^cAB^	129.62±11.22^bAB^	131.25±11.56^bB^	223.50±19.93^aAB^

A: 10.42 μg AFB_1_ kg^-1^; B: 25.71 μg AFB_1_ kg^-1^; C: 56.47 μg AFB_1_ kg^-1^.

*Results expressed as mean ± standard error. Means followed by different lower case letters in the same row differ significantly (P<0.05). Means followed by different upper case letters in the same column, within each parameter, differ significantly (P<0.05).

**Fig 1:**
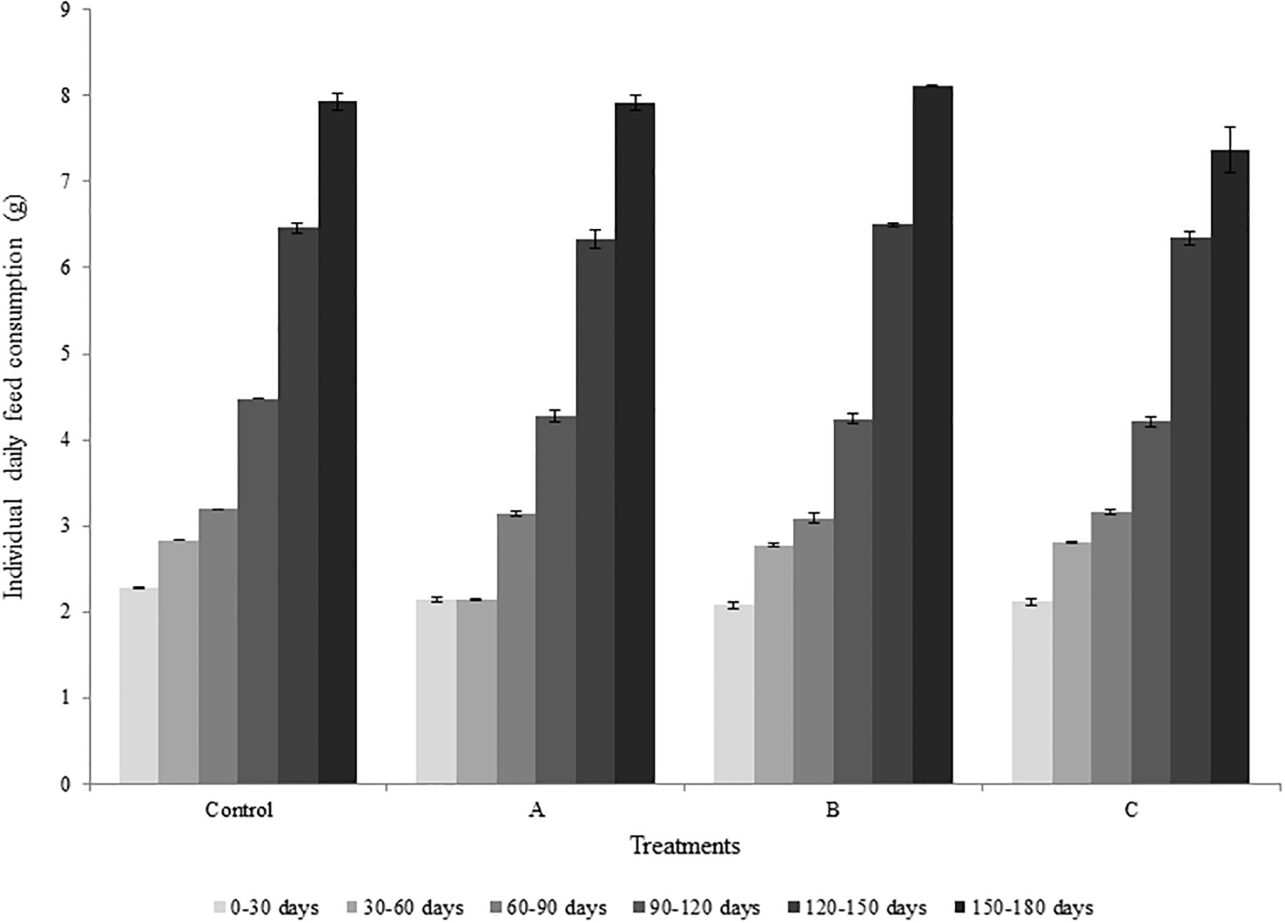
Individual daily feed consumption of matrinxã (*Brycon cephalus*) fish over the 180 days trial.

Fig 1 shows the daily dietary intake of matrinxã during the 180 days of exposure to aflatoxins. There were no differences among treatments (P > 0.05). The fish survival rate was 98.9% in the control treatment, 99.1% in treatment A, 99.8% in treatment B and 98.0% in treatment C.

The effects of aflatoxins on performance in several species of fish have already been studied (Table 5). In view of the work presented, it can be noted that there are, in fact, many differences in the susceptibility of fish species to aflatoxins. Abdelhamid [17] stated that catfish (order *Siluriformes*) are more resistant to aflatoxicosis than tilapia (*Oreochromis*). The mullet fish (*Mugil cephalus*) is very sensitive to AFB_1_, followed by carp (*Cyprinus carpio*), red tilapia (*Oreochromis* sp.) and Nile tilapia (*Oreochromis niloticus*), respectively [17]. The most species studies are sensitive to some effect of aflatoxins (Table 5). However, Huang et al. [30] reported that gibel carp (*Carassius auratus gibelio*) fed AFB_1_ for 24 weeks at 22.3 μg kg^-1^ and 1646.5 μg kg^-1^ were not affected for growth, mortality and feed conversion. Baglodi et al. [37] reported that survival, weight gain, length and feed conversion ratio were not affected in Indian carp (*Labeo rohita*) exposed to diets containing AFB_1_ at 50 μg kg^-1^, 100 μg kg^-1^ and 150 μg kg^-1^ for 130 days.

**Table 5:** Effects of aflatoxins on performance of several fish species.

Fish species	Aflatoxins in diets	Time of exposure	Effects	Reference
Channel catfish (*Ictalurus punctatus*)	10 mg AFB_1_ kg^-1^		Reduced growth	[38]
Nile tilapia (*Oreochromis niloticus*)	375; 752; 940; 1,500; 1,880 and 3,000 μg kg^-1^	25 days	Lower feed intake and growth rate	[39]
Nile tilapia (*Oreochromis niloticus*)	250; 2,500; 10,000 and 100,000 μg kg^-1^	8 weeks	Reduced weight gain above 2,500 μg kg^-1^ level Lower survival rate at the highest level	[40]
Nile tilapia (*Oreochromis niloticus*)	5; 115 μg kg^-1^		Lower survival rates, abnormal swimming, reduced appetite, blindness, body lesions	[41]
Nile tilapia (*Oreochromis niloticus*)	20 and 100 μg AFB_1_ kg^-1^	12 weeks	Reduced growth rate and weight gain at 100 μg kg^-1^ level	[42]
Nile tilapia (*Oreochromis niloticus*)	19; 85; 245; 638; 793 and 1,641 μg AFB_1_ kg^-1^	> 20 weeks	Lower growth, lower feed conversion; lower feed intake at 1,641 μg kg^-1^	[25]
Rainbow trout (*Oncorhynchus mykiss*)			Reduced growth rate and weight gain	[43]
White surgeon (*Huso huso*)	25; 50; 75 and 100 μg kg^-1^	3 months	Altered feed conversion and weight gain	[44]
White surgeon (*Huso huso*)	10 μg kg^-1^		Reduced feed intake, change in the swimming Hybrid sturgeon: decreased feed intake	[45], [46]
Red drum (*Sciaenops ocellatus*)	100; 250; 500; 1,000; 2,000; 3,000 and 5,000 μg kg^-1^	7 weeks	Decreased weight gain, survival rate and lower feed conversion	[47]
Yellow catfish (*Pelteobagrus fulvidraco*)	200; 500 and 1,000 μg kg^-1^	12 weeks	Lower survival rate, weight gain, growth rate and altered feed conversion	[48]
Jundiá (*Rhamdia quelen*)	41, 90 and 204 μg kg^-1^ 300, 757 and 1,177 μg kg^-1^	45 days	Lower levels of protein in muscle; lower weight and length gain	[49], [18]

The metabolic route for AFB_1_ in the liver involves demethylation to AFP_1_, reduction to aflatoxicol, epoxidation to AFB1-8,9-epoxide (AFBO) and hydroxylation to AFM, AFP_1_, AFQ_1_ or AFB_2a_. In general, phase I of the metabolism of aflatoxins converts the original molecules into more hydrophilic compounds by means of oxidation/reduction reactions. Phase II is characterized by conjugation of the original molecule or its metabolites with glutathione, glucuronides and sulfonides, with glutathione conjugation being considered the main route of detoxification which is catalyzed by glutathione-S-transferase [50].

In fact, the ability of a given animal to biotransform AFB_1_ into less toxic compounds influences its susceptibility. Thus, it can be inferred that the species that produces the more aflatoxicol are more sensitive to acute intoxications, since this compound can be reverted to AFB_1_ [24]. The lower survival rates of fish exposed to aflatoxins are due to damage to the immune system caused by the inhibition of protein synthesis resulting from the formation of adducts between AFBO and DNA or RNA [51].

The difference in susceptibility among species also appears to be related to the ability of hepatocytes to convert AFB_1_ to AFBO. Detoxification ability may also have an influence on susceptibility. As an example, the glutathione-S-transferase system in the liver can promote the formation of aflatoxin-glutathione conjugates, improving detoxification [51]. However, the glutathione-S-transferase system seems to be a less important detoxification pathway of aflatoxins in fish [35]. On the other hand, a fish that has two liver detoxification systems, such as glucuronyl transferase and sulfotransferase activity, will be less sensitive to the effects of toxic substances than those with only the glucuronyl transferase system [51].

Therefore, it is possible that a variety of metabolic enzymes in fish species is a potential mechanism for different aflatoxin sensitivities. In addition, there are variations among individuals in the rate of activation of aflatoxins in several species. Age and regions are also important factors that affect species resistance to AFB_1_. The AFBO conversion rate and its conjugation with glutathione are key parameters in interspecies and inter individual differences in sensitivity to the toxic effect of AFB_1_ [50]. In trout, glucuronidation represents the main pathway of phase II, whereas glutathione conjugates are often not detected as excreted metabolites [35].

In Brazil, fish farming has great social and economic relevance and contributes to the development of several activities because it is constantly growing within the agricultural sector [52]. It is known that feed is the item with the greatest impact on the final production costs in fish farming [53], and the quality of feed depends mainly on the sanitary control of the raw material, since corn, soybeans, rice and wheat are often contaminated by mycotoxin-producing fungi [54, 55, 56]. As shown in this study, long term consumption of aflatoxin-contaminated feed may impair fish performance, causing economic losses in fish production, besides the product becomes a potential risk to the consumers. Although the human contamination through the consumption of fish with aflatoxins residues is little studied, the accumulation in edible tissues is a possible phenomenon in several fish species [18, 19], playing a role in human health.

## Conclusion

Long term exposure of Matrinxã (*Brycon cephalus*) to dietary aflatoxins produces little or no residues of aflatoxin B_1_ in tissues, suggesting a resistance to aflatoxin B_1_ accumulation in edible parts. However, the chronic exposure to aflatoxins in the diet led to effects on weight and length indicating a susceptibility effect of aflatoxins over performance.

## Supporting information

S1 Fig Data for the individual daily feed consumption of matrinxã (*Brycon cephalus*) fish. Statistical analyses of Fig 1 data.
(DOCX)

S1 Table Data for aflatoxins levels in experimental diets. Statistical analyses of Table 2 data.
(DOCX)

S2 Table Data for aflatoxin B_1_ residues in liver and muscle of matrinxã (*Brycon cephalus*). Statistical analyses of Table 3 data.
(DOCX)

S3 Table Data for length and weight of matrinxã (*Brycon cephalus*) fish daily exposed to aflatoxins. Statistical analyses of Table 4 data.
(DOCX)
