# Correction: Effects of dietary aflatoxin B_1_ on accumulation and performance in matrinxã fish (*Brycon cephalus*)

**DOI:** 10.1371/journal.pone.0216194

**Published:** 2019-04-24

**Authors:** Carolina M. Bedoya-Serna, Euder C. Michelin, Marina M. Massocco, Lucas C. S. Carrion, Silvia H. S. Godoy, Cesar G. Lima, Paulo S. Ceccarelli, George S. Yasui, George E. Rottinghaus, Ricardo L. M. Sousa, Andrezza M. Fernandes

The following information is missing from the Funding statement: This study was supported by Conselho Nacional de Desenvolvimento Científico e Tecnológico (CNPq) [grant number 307391/2016-7].
