# Supplementary material for: Effects of dietary aflatoxin B1 on accumulation and performance in matrinxã fish (Brycon cephalus)
Source: PLoS One. 2018 Aug 8;13(8):e0201812. doi: 10.1371/journal.pone.0201812 (PMC6082536; doi:10.1371/journal.pone.0201812)
Supplement: S1 Fig — Statistical analyses of Fig 1 data. (DOCX) [file pone.0201812.s001.docx]

**S1_fig1**

**Data for the individual daily feed consumption of matrinxã (*Brycon cephalus*) fish.**

Statistical analyses of Fig 1 data.

MATRINXÃ consumption

Descriptive statistics: Individual daily consumption (g)

Aflatoxins: 0 µg/kg

Day Mean SE

30 2.2770 0.0028

60 2.8300 0.0000

90 3.2000 0.0000

120 4.4800 0.0000

150 6.4600 0.0524

180 7.9293 0.0912

Aflatoxins: 10 µg/kg

Day Mean SE

30 2.1356 0.0303

60 2.8115 0.0104

90 3.1531 0.0278

120 4.2803 0.0661

150 6.3290 0.1070

180 7.9190 0.0873

Aflatoxins: 20 µg/kg

Day Mean SE

30 2.0800 0.0359

60 2.7845 0.0250

90 3.0935 0.0644

120 4.2391 0.0582

150 6.5035 0.0203

180 8.1055 0.0145

Aflatoxins: 50 µg/kg

Day Mean SE

30 2.1233 0.0361

60 2.8124 0.0099

90 3.1573 0.0262

120 4.2138 0.0653

150 6.3523 0.0796

180 7.3670 0.2560
