# Supplementary material for: Effects of dietary aflatoxin B1 on accumulation and performance in matrinxã fish (Brycon cephalus)
Source: PLoS One. 2018 Aug 8;13(8):e0201812. doi: 10.1371/journal.pone.0201812 (PMC6082536; doi:10.1371/journal.pone.0201812)
Supplement: S1 Table — Statistical analyses of Table 2 data. (DOCX) [file pone.0201812.s002.docx]

**S1_table**

**Data for aflatoxins levels in experimental diets.** Statistical analyses of Table 2 data.

Dependent Variable: AFB1

Sum of

Source DF Squares Mean Square F Value Pr > F

Trat 2 16481.74766 8240.87383 91.74 <.0001

Error 36 3233.88652 89.83018

Corrected Total 38 19715.63418

R-Square Coeff Var Root MSE B1 Mean

0.835973 29.85525 9.477879 31.74610

The GLM Procedure

Level of --------------B1-------------

Trat N Mean Std Dev

A 15 10.4237333 3.7904760

B 9 25.7080000 5.6213176

C 15 56.6913333 14.0913979

Standard

Trat Estimate Error

A 10.4237 2.4472

B 25.7080 3.1593

C 56.6913 2.4472

**Tukey-Kramer Grouping for Trat Least Squares Means (Alpha=0.05)**

**LS-means with the same letter are not significantly different.**

**Trat Estimate**

**C 56.6913 A**

**B 25.7080 B**

**A 10.4237 C**

Dependent Variable: Total AF

Sum of

Source DF Squares Mean Square F Value Pr > F

Trat 2 18810.03209 9405.01604 74.35 <.0001

Error 36 4554.17452 126.50485

Corrected Total 38 23364.20661

R-Square Coeff Var Root MSE Totais Mean

0.805079 31.65720 11.24744 35.52885

The GLM Procedure

Level of ------------Totais-----------

Trat N Mean Std Dev

A 15 13.1464000 5.2503825

B 9 28.1111111 6.5484721

C 15 62.3619333 16.5295912

Standard

Trat Estimate Error

A 13.1464 2.9041

B 28.1111 3.7491

C 62.3619 2.9041

**Tukey-Kramer Grouping for Trat Least Squares Means (Alpha=0.05)**

**LS-means with the same letter are not significantly different.**

**Trat Estimate**

**C 62.3619 A**

**B 28.1111 B**

**A 13.1464 C**

>> DIFERENÇA SIGNIFICATIVA ENTRE AS MÉDIAS DAS TRÊS RAÇÕES
