# Supplementary material for: Effects of dietary aflatoxin B1 on accumulation and performance in matrinxã fish (Brycon cephalus)
Source: PLoS One. 2018 Aug 8;13(8):e0201812. doi: 10.1371/journal.pone.0201812 (PMC6082536; doi:10.1371/journal.pone.0201812)
Supplement: S2 Table — Statistical analyses of Table 3 data. (DOCX) [file pone.0201812.s003.docx]

**S2_table**

**Data for aflatoxin B1 residues in liver and muscle of matrinxã (*Brycon cephalus*).** Statistical analyses of Table 3 data.

Data Set WORK.MATRINXA_LIVER

Dependent Variable AFB1

Dose*Dia Least Squares Means

Standard

Dose Dia Estimate Error DF t Value Pr > |t|

10 30 0.07667 0.03331 27 2.30 0.0293

10 60 0.09333 0.03331 27 2.80 0.0093

10 90 0.07333 0.03331 27 2.20 0.0364

10 120 0.1800 0.03331 27 5.40 <.0001

10 150 0.3200 0.03331 27 9.61 <.0001

10 180 0.1533 0.03331 27 4.60 <.0001

20 30 0.1133 0.03331 27 3.40 0.0021

20 60 0.09667 0.03331 27 2.90 0.0073

20 90 0.1750 0.04079 27 4.29 0.0002

20 120 0.2667 0.03331 27 8.01 <.0001

20 150 0.3933 0.03331 27 11.81 <.0001

20 180 0.1400 0.03331 27 4.20 0.0003

50 30 0.4067 0.03331 27 12.21 <.0001

50 60 0.1667 0.03331 27 5.00 <.0001

50 90 0.2567 0.03331 27 7.71 <.0001

50 120 0.3100 0.04079 27 7.60 <.0001

50 150 0.4000 0.03331 27 12.01 <.0001

50 180 0.6050 0.04079 27 14.83 <.0001

Data Set WORK.MATRINXA_MUSCLE

Dependent Variable AFB1

Dose*Dia Least Squares Means

Standard

Dose Dia Estimate Error DF t Value Pr > |t|

10 30 0.03667 0.005212 30 7.04 <.0001

10 60 0.01667 0.005212 30 3.20 0.0033

10 90 0.01333 0.005212 30 2.56 0.0158

10 120 0.01000 0.005212 30 1.92 0.0646

10 150 0.03000 0.005212 30 5.76 <.0001

10 180 0.006667 0.005212 30 1.28 0.2106

20 30 0.02000 0.005212 30 3.84 0.0006

20 60 0.01000 0.005212 30 1.92 0.0646

20 90 0.01000 0.005212 30 1.92 0.0646

20 120 0.01000 0.005212 30 1.92 0.0646

20 150 0.03333 0.005212 30 6.40 <.0001

20 180 0.01000 0.005212 30 1.92 0.0646

50 30 0.03333 0.005212 30 6.40 <.0001

50 60 0.01667 0.005212 30 3.20 0.0033

50 90 0.02667 0.005212 30 5.12 <.0001

50 120 0.01667 0.005212 30 3.20 0.0033

50 150 0.05667 0.005212 30 10.87 <.0001

50 180 0.02667 0.005212 30 5.12 <.0001
