# Supplementary material for: Effects of dietary aflatoxin B1 on accumulation and performance in matrinxã fish (Brycon cephalus)
Source: PLoS One. 2018 Aug 8;13(8):e0201812. doi: 10.1371/journal.pone.0201812 (PMC6082536; doi:10.1371/journal.pone.0201812)
Supplement: S3 Table — Statistical analyses of Table 4 data. (DOCX) [file pone.0201812.s004.docx]

**S3_table**

**Data for length and weight of matrinxã (*Brycon cephalus*) fish daily exposed to aflatoxins.**

Statistical analyses of Table 4 data.

Data Set WORK.BIO_MATRINXA

Dependent Variable **LENGHT**

Covariance Structure Variance Components

Subject Effect Rep(Dose)

Type 3 Tests of Fixed Effects

Num Den

Effect DF DF F Value Pr > F

**Dose 3 84 33.86 <.0001**

**Dia 6 279 223.37 <.0001**

Dose*Dia 18 279 1.23 0.2393 n.s.

Tests of Effect Slices

Num Den

Effect Dose Dia DF DF F Value Pr > F

Dose*Dia 0 6 279 71.72 <.0001

Dose*Dia 10 6 279 54.11 <.0001

Dose*Dia 20 6 279 49.85 <.0001

Dose*Dia 50 6 279 52.50 <.0001

Tukey-Kramer Grouping for Dose*Dia Least Squares Means Slice (Alpha=0.05)

LS-means with the same letter are not significantly different.

Slice Dia Estimate

Dose 0 180 26.1500 A

Dose 0 150 22.2500 B

Dose 0 120 20.8462 B

Dose 0 90 18.8000 C

Dose 0 60 17.3333 D C

Dose 0 30 16.2870 D E

Dose 0 0 15.6000 E

Tukey-Kramer Grouping for Dose*Dia Least Squares Means Slice (Alpha=0.05)

LS-means with the same letter are not significantly different.

Slice Dia Estimate

Dose 10 180 23.8182 A

Dose 10 150 20.1875 B

Dose 10 120 18.6000 C B

Dose 10 90 16.6923 C D

Dose 10 60 15.8889 E D

Dose 10 30 15.2750 E D

Dose 10 0 14.6071 E

Tukey-Kramer Grouping for Dose*Dia Least Squares Means Slice (Alpha=0.05)

LS-means with the same letter are not significantly different.

Slice Dia Estimate

Dose 20 180 22.6000 A

Dose 20 150 20.5625 A

Dose 20 120 18.0000 B

Dose 20 90 17.1923 B

Dose 20 30 14.9792 C

Dose 20 60 14.6667 C

Dose 20 0 14.4000 C

Tukey-Kramer Grouping for Dose*Dia Least Squares Means Slice (Alpha=0.05)

LS-means with the same letter are not significantly different.

Slice Dia Estimate

Dose 50 180 23.7000 A

Dose 50 150 19.8125 B

Dose 50 120 19.3846 B

Dose 50 90 17.0000 C

Dose 50 60 15.7188 D C

Dose 50 30 15.5000 D C

Dose 50 0 14.4000 D

Data Set WORK.BIO_MATRINXA

Dependent Variable **WEIGHT**

Covariance Structure Variance Components

Subject Effect Rep(Dose)

Group Effect Dia

Covariance Parameter Estimates

Standard Z

Cov Parm Subject Group Estimate Error Value Pr > Z

Residual Rep(Dose) Dia 0 137.68 26.2549 5.24 <.0001

Residual Rep(Dose) Dia 30 261.26 40.3128 6.48 <.0001

Residual Rep(Dose) Dia 60 378.63 65.9110 5.74 <.0001

Residual Rep(Dose) Dia 90 687.94 137.59 5.00 <.0001

Residual Rep(Dose) Dia 120 1635.16 352.65 4.64 <.0001

Residual Rep(Dose) Dia 150 1069.53 285.84 3.74 <.0001

Residual Rep(Dose) Dia 180 3970.70 923.17 4.30 <.0001

Type 3 Tests of Fixed Effects

Num Den

Effect DF DF F Value Pr > F

Dose 3 84 17.94 <.0001

Dia 6 279 140.47 <.0001

Dose*Dia 18 279 1.98 0.0111

Tests of Effect Slices

Num Den

Effect Dose Dia DF DF F Value Pr > F

Dose*Dia 0 6 279 60.91 <.0001

Dose*Dia 10 6 279 29.83 <.0001

Dose*Dia 20 6 279 27.08 <.0001

Dose*Dia 50 6 279 30.10 <.0001

Tukey-Kramer Grouping for Dose*Dia Least Squares Means Slice (Alpha=0.05)

LS-means with the same letter are not significantly different.

Slice Dia Estimate

Dose 0 180 282.00 A

Dose 0 150 190.00 B

Dose 0 120 162.31 B

Dose 0 90 107.33 C

Dose 0 60 79.7222 D

Dose 0 30 63.3913 E D

Dose 0 0 50.8000 E

Tukey-Kramer Grouping for Dose*Dia Least Squares Means Slice (Alpha=0.05)

LS-means with the same letter are not significantly different.

Slice Dia Estimate

Dose 10 180 225.91 A

Dose 10 150 139.37 B

Dose 10 120 109.70 C B

Dose 10 90 80.0000 C D

Dose 10 60 59.4444 E D

Dose 10 30 53.5000 E F

Dose 10 0 42.5714 F

Tukey-Kramer Grouping for Dose*Dia Least Squares Means Slice (Alpha=0.05)

LS-means with the same letter are not significantly different.

Slice Dia Estimate

Dose 20 180 197.50 A

Dose 20 150 143.75 B A

Dose 20 120 110.00 B C

Dose 20 90 81.9231 C

Dose 20 30 51.5833 D

Dose 20 60 48.8889 D

Dose 20 0 43.0667 D

Tukey-Kramer Grouping for Dose*Dia Least Squares Means Slice (Alpha=0.05)

LS-means with the same letter are not significantly different.

Slice Dia Estimate

Dose 50 180 223.50 A

Dose 50 150 131.25 B

Dose 50 120 129.62 B

Dose 50 90 86.9231 C

Dose 50 60 66.8750 D C

Dose 50 30 57.5238 D E

Dose 50 0 45.8667 E

Tests of Effect Slices

Num Den

Effect Dose Dia DF DF F Value Pr > F

Dose*Dia 0 3 279 1.53 0.2073

Dose*Dia 30 3 279 2.39 0.0689

Dose*Dia 60 3 279 7.97 <.0001

Dose*Dia 90 3 279 3.28 0.0214

Dose*Dia 120 3 279 4.50 0.0042

Dose*Dia 150 3 279 5.23 0.0016

Dose*Dia 180 3 279 3.19 0.0240

Tukey-Kramer Grouping for Dose*Dia Least Squares Means Slice (Alpha=0.05)

LS-means with the same letter are not significantly different.

Slice Dose Estimate

Dia 0 0 50.8000 A

Dia 0 50 45.8667 A

Dia 0 20 43.0667 A

Dia 0 10 42.5714 A

Tukey-Kramer Grouping for Dose*Dia Least Squares Means Slice (Alpha=0.05)

LS-means with the same letter are not significantly different.

Slice Dose Estimate

Dia 30 0 63.3913 A

Dia 30 50 57.5238 A

Dia 30 10 53.5000 A

Dia 30 20 51.5833 A

+Tukey-Kramer Grouping for Dose*Dia Least Squares Means Slice (Alpha=0.05)

LS-means with the same letter are not significantly different.

Slice Dose Estimate

Dia 60 0 79.7222 A

Dia 60 50 66.8750 B A

Dia 60 10 59.4444 B C

Dia 60 20 48.8889 C

Tukey-Kramer Grouping for Dose*Dia Least Squares Means Slice (Alpha=0.05)

LS-means with the same letter are not significantly different.

Slice Dose Estimate

Dia 90 0 107.33 A

Dia 90 50 86.9231 B A

Dia 90 20 81.9231 B A

Dia 90 10 80.0000 B

Tukey-Kramer Grouping for Dose*Dia Least Squares Means Slice (Alpha=0.05)

LS-means with the same letter are not significantly different.

Slice Dose Estimate

Dia 120 0 162.31 A

Dia 120 50 129.62 B A

Dia 120 20 110.00 B

Dia 120 10 109.70 B

Tukey Grouping for Dose*Dia Least Squares Means Slice (Alpha=0.05)

LS-means with the same letter are not significantly different.

Slice Dose Estimate

Dia 150 0 190.00 A

Dia 150 20 143.75 B

Dia 150 10 139.37 B

Dia 150 50 131.25 B

Tukey-Kramer Grouping for Dose*Dia Least Squares Means Slice (Alpha=0.05)

LS-means with the same letter are not significantly different.

Slice Dose Estimate

Dia 180 0 282.00 A

Dia 180 10 225.91 B A

Dia 180 50 223.50 B A

Dia 180 20 197.50 B

Dose Least Squares Means

Standard

Dose Estimate Error DF t Value Pr > |t|

0 133.65 3.8966 84 34.30 <.0001

10 101.50 3.9254 84 25.86 <.0001

20 96.6731 3.9729 84 24.33 <.0001

50 105.94 3.9247 84 26.99 <.0001

Dia Least Squares Means

Standard

Dia Estimate Error DF t Value Pr > |t|

0 45.5762 1.5283 279 29.82 <.0001

30 56.4996 1.7275 279 32.71 <.0001

60 63.7326 2.3288 279 27.37 <.0001

90 89.0449 3.5761 279 24.90 <.0001

120 127.91 5.9358 279 21.55 <.0001

150 151.09 5.7812 279 26.14 <.0001

180 232.23 9.8494 279 23.58 <.0001

Dose*Dia Least Squares Means

Standard

Dose Dia Estimate Error DF t Value Pr > |t|

0 0 50.8000 3.0297 279 16.77 <.0001

0 30 63.3913 3.3703 279 18.81 <.0001

0 60 79.7222 4.5864 279 17.38 <.0001

0 90 107.33 6.7722 279 15.85 <.0001

0 120 162.31 11.2152 279 14.47 <.0001

0 150 190.00 11.5625 279 16.43 <.0001

0 180 282.00 19.9266 279 14.15 <.0001

10 0 42.5714 3.1360 279 13.58 <.0001

10 30 53.5000 3.6143 279 14.80 <.0001

10 60 59.4444 4.5864 279 12.96 <.0001

10 90 80.0000 7.2745 279 11.00 <.0001

10 120 109.70 12.7873 279 8.58 <.0001

10 150 139.37 11.5625 279 12.05 <.0001

10 180 225.91 18.9993 279 11.89 <.0001

20 0 43.0667 3.0297 279 14.22 <.0001

20 30 51.5833 3.2993 279 15.63 <.0001

20 60 48.8889 4.5864 279 10.66 <.0001

20 90 81.9231 7.2745 279 11.26 <.0001

20 120 110.00 12.1923 279 9.02 <.0001

20 150 143.75 11.5625 279 12.43 <.0001

20 180 197.50 19.9266 279 9.91 <.0001

50 0 45.8667 3.0297 279 15.14 <.0001

50 30 57.5238 3.5272 279 16.31 <.0001

50 60 66.8750 4.8646 279 13.75 <.0001

50 90 86.9231 7.2745 279 11.95 <.0001

50 120 129.62 11.2152 279 11.56 <.0001

50 150 131.25 11.5625 279 11.35 <.0001

50 180 223.50 19.9266 279 11.22 <.0001

Tests of Effect Slices

Num Den

Effect Dose Dia DF DF F Value Pr > F

Dose*Dia 0 3 279 2.07 0.1049

Dose*Dia 30 3 279 3.04 0.0295

Dose*Dia 60 3 279 9.06 <.0001

Dose*Dia 90 3 279 5.34 0.0014

Dose*Dia 120 3 279 7.63 <.0001

Dose*Dia 150 3 279 3.88 0.0097

Dose*Dia 180 3 279 9.35 <.0001

Tukey-Kramer Grouping for Dose*Dia Least Squares Means Slice (Alpha=0.05)

LS-means with the same letter are not significantly different.

Slice Dose Estimate

Dia 0 0 15.6000 A

Dia 0 10 14.6071 A

Dia 0 20 14.4000 A

Dia 0 50 14.4000 A

Tukey-Kramer Grouping for Dose*Dia Least Squares Means Slice (Alpha=0.05)

LS-means with the same letter are not significantly different.

Slice Dose Estimate

Dia 30 0 16.2870 A

Dia 30 50 15.5000 B A

Dia 30 10 15.2750 B A

Dia 30 20 14.9792 B

Tukey-Kramer Grouping for Dose*Dia Least Squares Means Slice (Alpha=0.05)

LS-means with the same letter are not significantly different.

Slice Dose Estimate

Dia 60 0 17.3333 A

Dia 60 10 15.8889 B

Dia 60 50 15.7188 C B

Dia 60 20 14.6667 C

Tukey-Kramer Grouping for Dose*Dia Least Squares Means Slice (Alpha=0.05)

LS-means with the same letter are not significantly different.

Slice Dose Estimate

Dia 90 0 18.8000 A

Dia 90 20 17.1923 B

Dia 90 50 17.0000 B

Dia 90 10 16.6923 B

Tukey-Kramer Grouping for Dose*Dia Least Squares Means Slice (Alpha=0.05)

LS-means with the same letter are not significantly different.

Slice Dose Estimate

Dia 120 0 20.8462 A

Dia 120 50 19.3846 B A

Dia 120 10 18.6000 B

Dia 120 20 18.0000 B

Tukey Grouping for Dose*Dia Least Squares Means Slice (Alpha=0.05)

LS-means with the same letter are not significantly different.

Slice Dose Estimate

Dia 150 0 22.2500 A

Dia 150 20 20.5625 B A

Dia 150 10 20.1875 B

Dia 150 50 19.8125 B

Tukey-Kramer Grouping for Dose*Dia Least Squares Means Slice (Alpha=0.05)

LS-means with the same letter are not significantly different.

Slice Dose Estimate

Dia 180 0 26.1500 A

Dia 180 10 23.8182 B

Dia 180 50 23.7000 B

Dia 180 20 22.6000 B

Dose Least Squares Means

Standard

Dose Estimate Error DF t Value Pr > |t|

0 19.6095 0.1693 84 115.80 <.0001

10 17.8670 0.1748 84 102.23 <.0001

20 17.4858 0.1735 84 100.80 <.0001

50 17.9308 0.1721 84 104.18 <.0001

Dia Least Squares Means

Standard

Dia Estimate Error DF t Value Pr > |t|

0 14.7518 0.1781 279 82.81 <.0001

30 15.5103 0.1504 279 103.10 <.0001

60 15.9019 0.1632 279 97.43 <.0001

90 17.4212 0.2142 279 81.32 <.0001

120 19.2077 0.2716 279 70.73 <.0001

150 20.7031 0.2665 279 77.67 <.0001

180 24.0670 0.3042 279 79.11 <.0001

Dose*Dia Least Squares Means

Standard

Dose Dia Estimate Error DF t Value Pr > |t|

0 0 15.6000 0.3531 279 44.17 <.0001

0 30 16.2870 0.2935 279 55.49 <.0001

0 60 17.3333 0.3215 279 53.92 <.0001

0 90 18.8000 0.4057 279 46.34 <.0001

0 120 20.8462 0.5131 279 40.63 <.0001

0 150 22.2500 0.5331 279 41.74 <.0001

0 180 26.1500 0.6155 279 42.49 <.0001

10 0 14.6071 0.3655 279 39.96 <.0001

10 30 15.2750 0.3148 279 48.53 <.0001

10 60 15.8889 0.3215 279 49.43 <.0001

10 90 16.6923 0.4358 279 38.30 <.0001

10 120 18.6000 0.5850 279 31.79 <.0001

10 150 20.1875 0.5331 279 37.87 <.0001

10 180 23.8182 0.5868 279 40.59 <.0001

20 0 14.4000 0.3531 279 40.78 <.0001

20 30 14.9792 0.2873 279 52.13 <.0001

20 60 14.6667 0.3215 279 45.63 <.0001

20 90 17.1923 0.4358 279 39.45 <.0001

20 120 18.0000 0.5578 279 32.27 <.0001

20 150 20.5625 0.5331 279 38.57 <.0001

20 180 22.6000 0.6155 279 36.72 <.0001

50 0 14.4000 0.3531 279 40.78 <.0001

50 30 15.5000 0.3072 279 50.46 <.0001

50 60 15.7188 0.3410 279 46.10 <.0001

50 90 17.0000 0.4358 279 39.01 <.0001

50 120 19.3846 0.5131 279 37.78 <.0001

50 150 19.8125 0.5331 279 37.17 <.0001

50 180 23.7000 0.6155 279 38.51 <.0001
